# Epidemiology of Neuroendocrine Neoplasms and Results of Their Treatment with [^177^Lu]Lu-DOTA-TATE or [^177^Lu]Lu-DOTA-TATE and [^90^Y]Y-DOTA-TATE—A Six-Year Experience in High-Reference Polish Neuroendocrine Neoplasm Center

**DOI:** 10.3390/cancers15225466

**Published:** 2023-11-18

**Authors:** Adam Daniel Durma, Marek Saracyn, Maciej Kołodziej, Katarzyna Jóźwik-Plebanek, Beata Dmochowska, Waldemar Kapusta, Wawrzyniec Żmudzki, Adrianna Mróz, Beata Kos-Kudła, Grzegorz Kamiński

**Affiliations:** 1 Department of Endocrinology and Radioisotope Therapy, Military Institute of Medicine—National Research Institute, 04-141 Warsaw, Poland; 2 Department of Endocrinology and Neuroendocrine Tumors, Department of Pathophysiology and Endocrinology in Zabrze, Medical University of Silesia, 40-055 Katowice, Poland

**Keywords:** neuroendocrine neoplasms, NEN, epidemiology, incidence, RLT, PRRT, ^177^Lu, ^90^Y

## Abstract

**Simple Summary:**

Neuroendocrine neoplasms (NENs) are tumors originating from neuroendocrine cells, with increasing global incidence and prevalence. Radioligand therapy (RLT) with beta-emitting radioisotopes is an effective and relatively safe treatment, surpassing other pharmacotherapies in tolerability. A study by Poland’s top radioisotope-treatment center analyzed 167 patients over 66 months, administering 479 RLT radioisotope doses. Non-functioning G2 NENs with a mean Ki-67 of 6.05% were predominant, often originating in the pancreas. Post-RLT disease stabilization occurred in 69.46%, partial regression in 20.36%, complete regression in 0.60%, and progression in 9.58% of patients. Long-term follow-up (median 29.8 months) revealed stabilization in 55.56% of patients, progression in 26.85% of patients, and a 17.59% mortality rate. Median PFS and OS were 29.3 and 34.0 months, respectively. Thus, the study showed that RLT lead to disease stabilization in over half of the patients with progressive disease in long-term observation. Poland’s coordinated NEN treatment in high-reference centers ensures consistent patient care.

**Abstract:**

Neuroendocrine neoplasms (NENs) are a group of neoplasms arising from neuroendocrine cells. The worldwide incidence and prevalence of the NENs are estimated to be 6/100,000 and 35/100,000, respectively. Those numbers are increasing every decade, requiring higher and higher diagnosis and treatment costs. Radioligand therapy (RLT) using beta-emitting radioisotopes is an efficient and relatively safe method of treatment, typically used as a second-line treatment. RLT tolerability is higher than other available pharmacotherapies (chemotherapy or tyrosine kinase inhibitors). Recent studies show an increase in overall survival among patients treated with RLT. The present study aimed to learn the epidemiology of NENs in Poland and assess the effectiveness of RLT in a high-reference center. A prospective analysis of 167 patients treated with RLT in one of Poland’s highest-reference NEN centers was performed. The analysis covered 66 months of observation (1 December 2017–30 May 2023), during which 479 RLT single administrations of radioisotope were given. The standard procedure was to give four courses of [^177^Lu]Lu-DOTA-TATE alone, or tandem therapy—[^177^Lu]Lu-DOTA-TATE and [^90^Y]Y-DOTA-TATE. Grading analysis showed that most patients had non-functioning G2 NEN with a mean Ki-67 of 6.05% (SD ± 6.41). The most common primary tumor location was the pancreas. Over two-thirds of patients did undergo surgery due to primary tumors or distant metastases. The majority of patients were using lanreotide as a chronically injected somatostatin analog. Median progression-free survival (PFS) on somatostatin analogs was 21.0 (IQR = 29.0) months. Directly after the last course of RLT, disease stabilization was noted in 69.46% of patients, partial regression was noted in 20.36% of patients, complete regression was noted in 0.60% of patients, and progression was noted in 9.58% of patients. In long-term follow-up, the median observation time among patients who underwent four treatment cycles (n = 108) was 29.8 (IQR = 23.9) months. Stabilization of the disease was observed in 55.56% of the patients and progression was observed in 26.85% of the patients, while 17.59% of patients died. Median PFS was 29.3 (IQR 23.9), and the median OS was 34.0 months (IQR 16.0). The mean age of NEN diagnosis is the sixth decade of life. It takes almost three years from NEN diagnosis to the start of RLT. In long-term observation, RLT leads to disease stabilization in over half of the patients with progressive disease. No differences in PFS or OS depend on the radioisotope used for RLT. In Poland, organized coordination of NEN treatment in high-reference centers ensures the continuity of patient care.

## 1. Introduction

Neuroendocrine neoplasms (NENs) are a heterogeneous group of neoplasms arising from specialized cells known as neuroendocrine cells [1]. Those cells have features similar to nerve- and hormone-producing cells. Despite having similar embryonic origin, neoplasms can be various in the context of the function, location, course, and outcome [2,3]. Worldwide, the most common location of neuroendocrine tumors is the small intestine. Next, neoplasms are found in the pancreas and other parts of the gastrointestinal tract [4]. In many cases, the primary location of the neoplasm is unknown, and the disease is found only due to local or distant metastases [5]. Previous statistics show that this type of neoplasm is present in 10–20% of all NEN cases [6,7].

Radioligand therapy (RLT), previously called peptide receptor radionuclide therapy (PRRT), is usually used as a second-line treatment. However, in inoperable or disseminated cases, it can be used as a first-line treatment [4,8,9]. RLT qualification is possible in NEN grades 1 (G1), 2 (G2), and 3 (G3); these are cases with confirmed somatostatin receptors expression in [^99m^Tc]-scintigraphy or [^68^Ga]Ga-PET/CT [4,10]. Preoperative studies with [^68^Ga]Ga-PET/CT are crucial for stratifying patients who are suitable for radioligand treatment. Despite PET/CT being more expensive and less available than scintigraphy, it has significantly higher sensitivity and image resolution [11,12].

Nowadays, there are two most-used “types” of the therapy: lutetium alone—[^177^Lu]Lu-DOTA-TATE; “tandem therapy”—[^177^Lu]Lu-DOTA-TATE and [^90^Y]Y-DOTA-TATE mixed. The usefulness of [^90^Y]Y-DOTA-TATE is still questionable due to the possible high number of adverse events. However, many studies confirmed the high utility and tumor mass reduction during the treatment [13,14]. There are ongoing discussions about the most appropriate type of treatment. Some data advocates for using lutetium only; however, others are showing that “tandem therapy” is just as safe and is more efficient than lutetium alone [15].

The epidemiological data show that the annual incidence of NEN is estimated at 5.86 per 100,000 persons/year; this value continues to increase [16,17]. The prevalence is estimated at 35/100,000 but may be considerably higher due to the occurrence of silent, non-functioning tumors [18]. Most data are retrospective; however, establishing a sizeable populational study could be difficult due to the relative rarity of the disease.

One key criterion for NEN division is the tumor grade assessed, thanks to using the Ki-67 proliferation index. The index describes several division figures in 10 large fields of view during microscopic assessment [19]. The Ki-67 index plays a crucial role in the preoperative assessment of treatment and serves as an independent prognostic marker for treatment outcomes. The World Health Organization’s Ki-67 labeling scheme provides a reliable basis for accurately grading endoscopic ultrasound fine-needle aspiration (EUS-FNA) samples of neuroendocrine neoplasms. This assessment aids in making reliable patient care and treatment decisions with a relatively low margin of error [20,21]. It is also important to note that tumor grading (Ki-67 index) could be determined through EUS-FNA in only 20% of pancreatic NENs while using tissue acquisition provides results in almost 70% of cases [22,23]. Moreover, the use of new markers of NEN—such as death-domain-associated protein (DAXX), α-thalassemia/mental retardation X-linked (ATRX) chromatin remodeling gene mutations, or alternative lengthening of telomeres (ALT) activation—could provide more detailed information about prognosis and offer a more accurate prediction of disease treatment [24]. The Ki-67 index is especially relevant for pancreatic NENs smaller than 20 mm in size, which can be under active surveillance instead of resorting to surgery.

Chromogranin is a non-specific antigen, which can be helpful in the diagnosis of the disease or in treatment; however, due to a lack of worldwide standardization, the method still needs to be fully used. Nevertheless, it remains a good predictor of prognosis and treatment outcomes [25,26].

While the first line of NEN treatment is surgery, besides the observation of asymptomatic and non-functioning tumors, the most up-to-date guidelines advocate for using RLT as a second line of G1 and G2 NEN treatment (or first, in cases of inoperable tumors)—this was previously chemotherapy [4,27]. Another potential treatment option includes local procedures such as endoscopic-ultrasound-guided radiofrequency ablation (EUS-RFA), endoscopic-ultrasound-guided ethanol ablation (EUS-EA), or the administration of radioisotopes directly to the arteries supplying the tumor or its liver metastases [28,29,30,31]. These minimally invasive methods offer relatively high safety and efficacy, particularly in pancreatic NENs. They could serve as a viable alternative to surgery for treating low-grade tumors, especially in patients with contraindications for standard treatment methods.

However, the abovementioned chemotherapy remains one of the best therapeutic options in some G3 NEN and neuroendocrine carcinomas (NECs). Practitioners must remember to individualize every therapy and tailor it to their patient. Chemotherapy can also be indicated for G1 and G2 NEN patients, who have no surgical options, for patients in whom RLT failed, and for those in whom RLT is contraindicated. Chemotherapy in well-differentiated G1/G2/G3 NEN usually does not improve significantly and can cause many adverse events and deterioration of the quality of life. Recommended regimens for chemotherapy are mostly two-component approaches, i.e., streptozocin (STZ) with 5-fluorouracil (5-FU) or doxorubicin (DOX); cisplatin (P) with etoposide (E); or capecitabine with temozolomide (CAPTEM) [4,32,33,34,35]. It is essential to note that local drug availability, comorbidities, and patient clinical conditions and expectations can also limit this kind of therapy.

The primary aim of the present study was to analyze the epidemiology and outcomes of RLT for NEN patients in one of the biggest ENETS-certified centers in Poland. The secondary aim was to evaluate the usefulness of chromogranin A as a marker of treatment outcomes.

## 2. Materials and Methods

Based on data from six years of single-centered prospective observation, we analyzed patients’ epidemiological details and outcomes of radioligand therapy, measured according to progression-free survival (PFS) and overall survival (OS) and indirectly by CgA concentrations. The Author’s Center is a part of the National Center of Excellence in treating neuroendocrine neoplasms (ENETS) and was the only clinic in the country with an uninterrupted possibility of delivering radioligand therapy in the analyzed period. Thanks to cooperation with other national centers, which served as local hubs for treating neuroendocrine neoplasms, it was possible to collect a vast amount of data and prepare the following analysis.

In the observation time (66 months, from December 2017 to May 2023), 167 patients qualified for radioligand therapy. They underwent RLT cycles in the Department of Endocrinology and Radioisotope Therapy of the Military Institute of Medicine—National Research Institute, Warsaw, Poland. The study was conducted in accordance with the Declaration of Helsinki and was approved by the local Ethics Committee, Protocol Code 154/17 (date of approval—15 December 2017). The total number of radioisotope administrations was 479. A subgroup of 127 patients who underwent exactly four courses of treatment was taken for a detailed assessment of treatment outcomes. In this subgroup, 13 patients died; in 28 patients, progression was observed; 67 patients remained stable; the statuses of 19 patients were unknown. Due to the length of this manuscript, we have not included an assessment of RLT complications; these will be presented and discussed in detail in an upcoming publication.

### 2.1. Treatment Protocol

Patients received RLT treatment in standard 4-course protocols. Patients received lutetium (7.4 GBq of [^177^Lu]Lu-DOTA-TATE) (LutaPol^®^, Polatom, Otwock, Poland) or tandem therapy (1.85 GBq [^90^Y]Y-DOTA-TATE +1.85 GBq + [^177^Lu]Lu-DOTA-TATE) (ItraPol^®^, Polatom, Otwock, Poland and LutaPol^®^, Polatom, Otwock, Poland). During 8–12-week intervals, long-lasting somatostatin analogs were administered: lanreotide (120 mg) or octreotide (30 mg) every four weeks. Intravenous nephroprotection using amino acids (Nephrotec^®^, Fresenius Kabi, Poland) was administrated with the center protocol. On the day of radioisotope administration, an infusion of 1000 mL was started 30 to 45 min before radioisotope infusion through the separate venal port, and another 500 mL infusion of amino acids was started a day after the treatment. Along with nephroprotection, ondansetron was given in the summary dose of 8–12 mg (depending on the gastrointestinal side effects reported by the patients). The standard protocol of 4 RLT courses was discontinued if complications, adverse events, or lack of consent for continuing the therapy occurred.

### 2.2. Statistical Analysis

Statistical analysis was performed using Microsoft Office 2021 Professional Package (Excel) and IBM SPSS (v29 2022). The Shapiro–Wilk test was conducted to verify whether the results met the standard distribution rules. Results with normal distribution were presented as means (M) and standard deviations (SD), and, in the case of non-normal distribution, as medians (Med.) and interquartile ranges (IQR). The Mann–Whitney U test was used to analyze dependencies between subgroups treated with different radioisotopes. Wilcoxon signed-rank test was used to compare the CgA results, with a significance level of <0.05.

### 2.3. Laboratory Evaluation

Regarding chromogranin (CgA), venous blood samples were taken during fasting, between 07:30 and 08:30 a.m. They were collected with the BD Vacutainer Tests in the Department of Endocrinology and Radioisotope Therapy and analyzed in the Department of Medical Diagnostics (Military Institute of Medicine—National Research Institute). The parameter was performed using LDN Company ELISA assays (Germany). The reference range for CgA was 19–100 ng/mL, and the sensitivity for this parameter was 1.4 µg/L.

## 3. Results

### 3.1. Gender and Age

Of the 167 patients, 51.9% (n = 85) were female, and 49.1% (n = 82) were male. The mean age of the patient was 59.9 years old, with a standard deviation (SD) of ± 12.36. In the female subgroup, the mean age at first treatment was 59.9 ± 12.85, and for males was 60.01 ± 11.83. The group presented normal distribution, in both female and male subgroups. The mean number of single radioligand administrations given to the patients was 3.72 ± 1.02. The mean age of NEN diagnosis in the study group was 57.23 ± 12.69. In the female subgroup, the NEN was diagnosed at 56.95 ± 13.51; in the male group, the age was 57.51 ± 11.81. Retrospectively analyzed median time (in months) from the diagnosis of NEN to RLT was 21.0 (IQR = 29.0), so the PFS on previous treatment (SSA) can be considered as above. The detailed data described above are presented in Table 1.

### 3.2. NEN Grading and Ki-67

**Figure 1:**
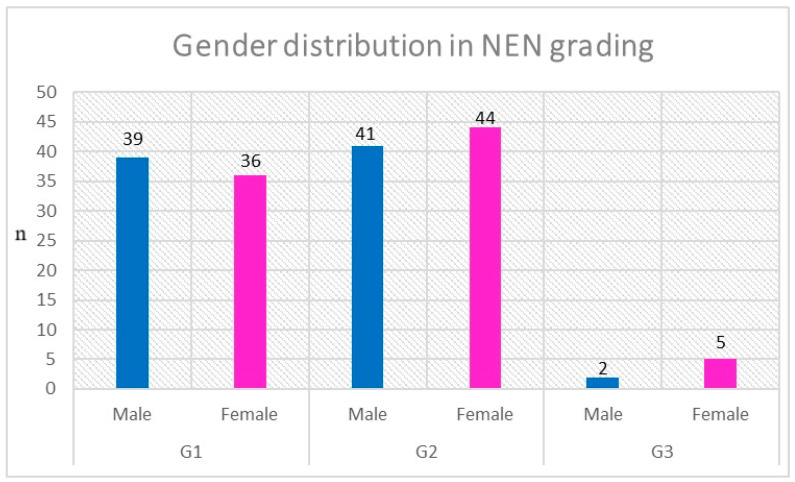
Gender distribution in NEN grading subgroups.

Among the study group, 44.91% (n = 75) patients had histological confirmation of G1 NEN, and 50.9% (n = 85) were diagnosed with G2 NEN. Only 4.19% (n = 7) patients were diagnosed with NEN G3. The difference in gender distribution was only noticeable in G3 NEN (2 vs. 5), although the results cannot be considered statistically significant due to the small size of the group. All patients’ mean proliferation index (Ki-67) was 6.05 ± 6.41. In the female subgroup, the Ki-67 index was higher than in the male subgroup (6.39 versus 5.70). The index range in both subgroups was the same and was 1–30%. The detailed data described above are presented in Figure 1.

### 3.3. Tumor Location and Functionality

**Figure 2:**
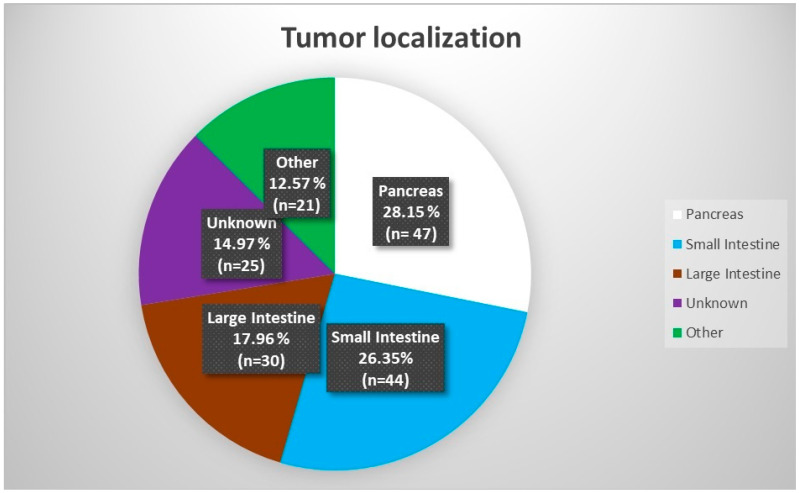
Distribution of primary tumor localization.

**Figure 3:**
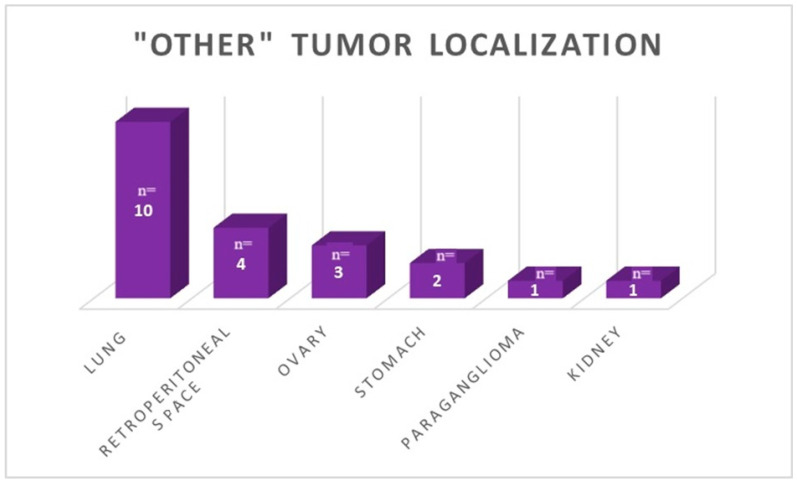
Distribution of primary tumor localization in locations other than GEP-NEN.

In the study group, the most common localization of the primary tumor was the pancreas, accounting for 28.14% (n = 47). Smaller numbers were observed for the following: small intestine—26.35% (n = 44); large intestine—17.96% (n = 30). The unknown localization of NEN was confirmed in 14.97% of cases (n = 25). Other than gastroenteropancreatic (non-GEP), primary tumor localization was confirmed in 21 cases (12.57%). The most common non-GEP localizations were the lungs (n = 10), the retroperitoneal space (n = 4), and the ovaries (n = 3). In our group, they were also found in single cases of scattered paraganglioma (n = 1) and kidney neuroendocrine tumor (n = 1). In 65.23% (n = 109) of cases, no functional activity of neoplasm was confirmed. In 28.74% (n = 48) of patients, clinical symptoms of carcinoid syndrome were observed—flushes, diarrhea, telangiectasias, or tachycardia. A proportion of 2.39% (n = 4) had histological, clinical, and laboratory confirmation of gastrinoma, while 1.20% (n = 2) were diagnosed with glucagonoma. Interestingly, both glucagonoma cases were initially diagnosed in dermatology departments during the diagnostic of necrolytic migratory erythema. In patients treated with RLT in our group, there were casual (n = 1) cases of insulinoma and tumors secreting growth hormone (GH), parathyroid hormone-related protein (PTHrP), and paraganglioma, as mentioned above. Before the first radioisotope administration, chromogranin A (CgA) concentrations were analyzed. The normal range for the CgA was 19–100 ng/mL; initially, in 41.8% of patients, the results were in the normal range. In the study subgroup with complete laboratory tests (n = 122), the median CgA concentration before the treatment was 133.1 ng/mL (IQR = 359.4). For the carcinoid subgroup (n = 33), which was intentionally separated from the group of functioning tumors, the median CgA concentration was 466.0 ng/mL (IQR = 5050.6). The non-functioning (n = 79) subgroup presented a median CgA concentration of 96.5 ng/mL (IQR = 240.1). After the treatment, the median CgA was 102.9 ng/mL (IQR = 395.0); this was 383.5 ng/mL (IQR= 7840.8) in the carcinoid subgroup, and 82.8 (IQR = 187.6) in the non-functioning subgroup. So, RLT caused a statistically significant decrease in CgA concentration in the study group (n = 122) and the non-functioning subgroup (n = 79) (*p*-value 0.034 and 0.018, respectively). In the carcinoid subgroup, CgA concentration was not a significant predictive parameter. The data described above are presented in Figure 2 and Figure 3 and Table 2.

### 3.4. Advancement and Radioisotope

**Figure 4:**
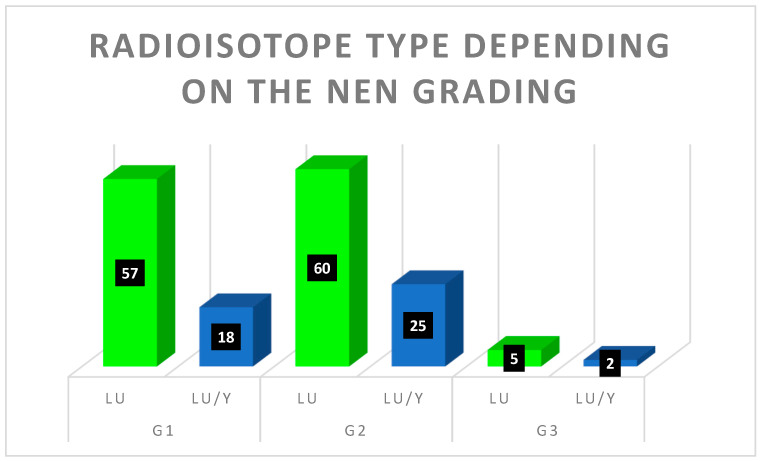
Radioisotope distribution in different NEN grading (in black boxes: number of patients).

The qualification for RLT was made by a multidisciplinary and multicenter team composed of endocrinologists, nuclear medicine specialists, oncologists, radiologists, and surgeons (Multidisciplinary Tumor Board of the ENETS Center of Excellence). One of the most important criteria was disease advancement under current medication confirmed in functional (^99m^Tc-scinitgraphy or [^68^Ga]Ga-PET/CT) as well as in anatomical imaging (CT/MRI). Most cases—95% (n = 195)—were patients with distant NEN metastases. The most common location for metastases was the liver. The local metastases, accounting for 4% (n = 6), had primary tumor growth and local nodules. The primary location—1% (n = 2)—was noted cases where only the primary tumor had enlarged significantly. Patients received standard activity of beta-emitting radioisotopes (7.4 GBq Lutetium-177 as [^177^Lu]Lu-DOTA-TATE or tandem therapy; 1.85 + 1.85 GBq of Lutetium-177 + Yttrium-90 as [^177^Lu]Lu-DOTA-TATE/[^90^Y]Y-DOTA-TATE). A radioisotope of lutetium was administrated in 73% of patients (122), while tandem therapy was given to 27% of patients (n = 45) A detailed distribution of the radioisotope used, correlated with NEN grading, has been presented in Figure 4.

### 3.5. Previous Surgery and Chemotherapy

Before RLT, 69% of patients (n = 116) underwent surgical operations of primary tumors and operable metastases. Disqualification from the surgery was made in 31% of patients (n = 51). The most common reason for non-surgical NEN was the unknown primary location (n = 20) and pancreatic location of the tumor (n = 20). Small intestine localization (duodenal) was the reason in four cases; other inoperable locations were confirmed in seven patients. The mean age of patients with inoperable tumors was 61.8 ± 10.23. The gender distribution of inoperable cases was almost equal (26 males vs. 25 females).

**Figure 5:**
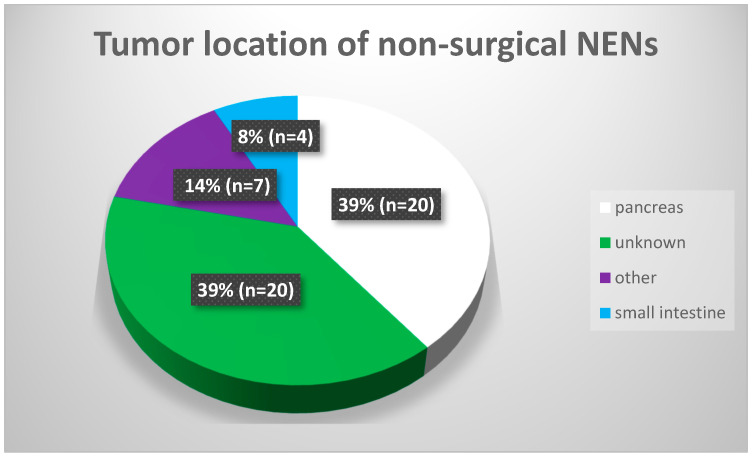
Non-surgical NEN tumor location.

Chemotherapy before RLT was given for a relatively high number of patients—21% (n = 35). In three cases, chemotherapy was introduced for the treatment of other malignant neoplasms (mucosa-associated lymphoid tissue lymphoma = MALToma, breast cancer, large intestine cancer). However, 32 patients received targeted chemotherapy for NEN treatment in many schemes. Surprisingly, chemotherapy was given to 10 NEN G1 patients, 19 NEN G2, and 3 NEN G3 patients, accounting for 13.33%, 22.35%, and 42.86% of their grading subgroups, respectively. One patient underwent the previous two (etoposide + cisplatin followed by 5-fluorouracil + leucovorin), and one patient underwent three different schemes of chemotherapy (etoposide + cisplatin followed by docetaxel followed by capecitabine + temozolomide) before RLT. We could not find documentation of chemotherapy (UNK) having been used in four patients. The detailed distribution of chemotherapy is presented in Figure 5 and Table 3 and Table 4.

### 3.6. Somatostatin Analogues

**Figure 6:**
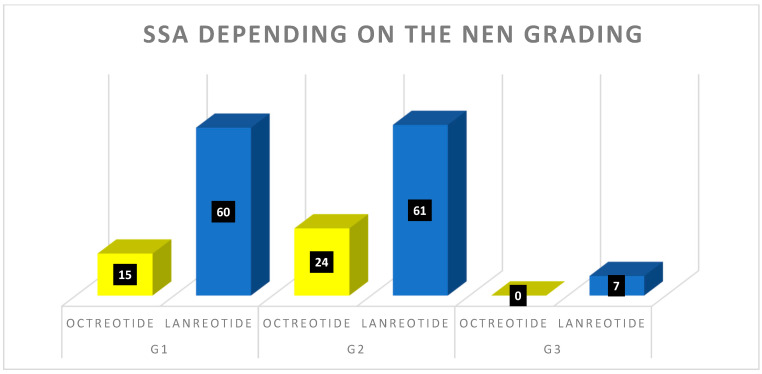
Detailed distribution of somatostatin analogs used in the study group (in black boxes: number of patients).

Initially, all 167 patients were taking long-lasting somatostatin analogs (SSA). Almost three-quarters of patients—74% (n = 123)—received monthly injections of lanreotide 120 mg as auto-gel. Only 26% (n = 44) of patients took octreotide at a monthly dose of 30 mg. Lanreotide predominance was the highest in cases of G3 NENs, where any patient received octreotide (7 vs. 0). In 12 patients, somatostatin analog was changed during the treatment. Four patients switched from lanreotide for octreotide, while eight changed from octreotide to lanreotide. In two patients, the therapy was stopped—one (male) suffered a subjectively unacceptable number of gastric adverse events, and the other (female) was suffering from subcutaneous abscesses. Despite the recommendation to change the SSA or the place of administration, she declined the possibility of returning to the treatment. Figure 6 presents the SSA administration details.

### 3.7. Comorbidities

Concomitant diseases were analyzed in glucose metabolism disorders, hypertension, and hyperlipidemia. Most patients (n = 92) did not have diabetes diagnosed before the treatment (55.09%). In 13.77% (n = 23) and 31.14% (n = 52) patients, prediabetes and diabetes were diagnosed, respectively.

Hypertension was diagnosed in 50.89% of patients (n = 85), while 49.11% of individuals (n = 82) did not have blood pressure elevation.

In 36.52% (n = 61) of patients, hyperlipidemia was present; elevated serum concentration of low-density lipoproteins (LDL), triglycerides (TG), or decreased concentration of high-density lipoproteins (HDL) was confirmed. Over 63.47% of patients (n = 106) did not present any of the abovementioned cholesterol metabolism disorders.

In the study group, 28.74% (n = 48) of patients had no metabolic or cardiovascular disease diagnosed before RLT. The subgroup of 17.37% (n = 29) of patients had only one factor, 23.95% (n = 40) of patients had two factors, and 29.94% (n = 50) of patients had all three factors.

**Figure 7:**
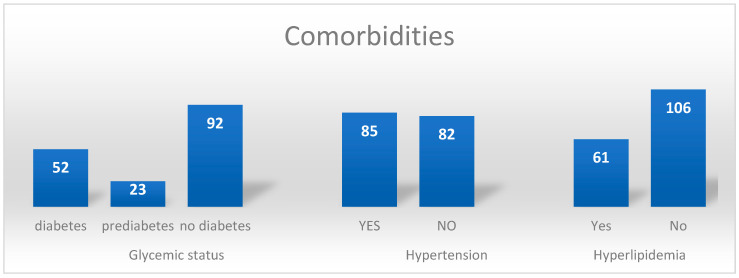
Details of comorbidities (=metabolic risk factors) in the study group (white text—number of patients).

Data regarding the most common metabolic diseases are presented in Figure 7.

### 3.8. Regions of Referral

**Figure 8:**
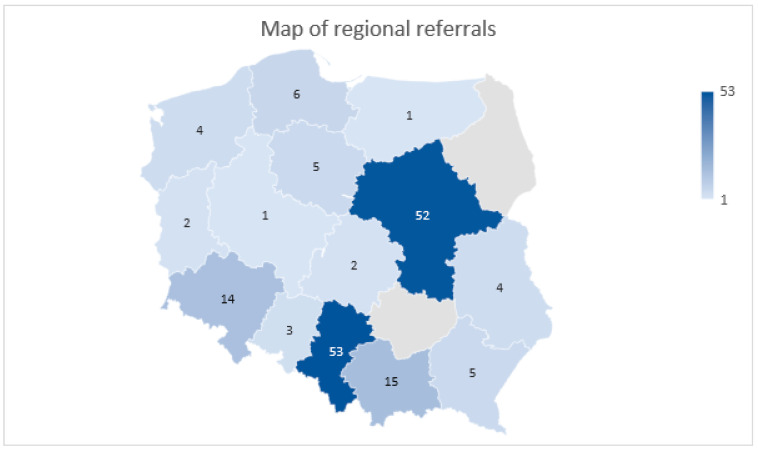
Map of Poland with marked referral regions. Number of patients referred from the center located in this region.

Patients in the study group were qualified for RLT in accordance with all Polish endocrinological and oncological centers dealing with NENs. The map in Figure 8 is the referral distribution of qualified patients who underwent RLT in our center.

### 3.9. Treatment Outcomes

In the observation directly after the last course of RLT, we observed disease stabilization in 69.46% of patients, partial regression in 20.36% of patients, complete regression in 0.60% of patients, and progression in 9.58% of patients.

For the precise assessment of the treatment outcomes, 127 patients who received only four courses (standard) of RLT were selected.

In follow-up, after the RLT (median observation time = 29.8 months; IQR = 23.9; n = 108), disease stabilization or partial regression was confirmed in 55.56% of patients, progression was confirmed in 26.85% of patients, and only 17.59% of patients died.

**Figure 9:**
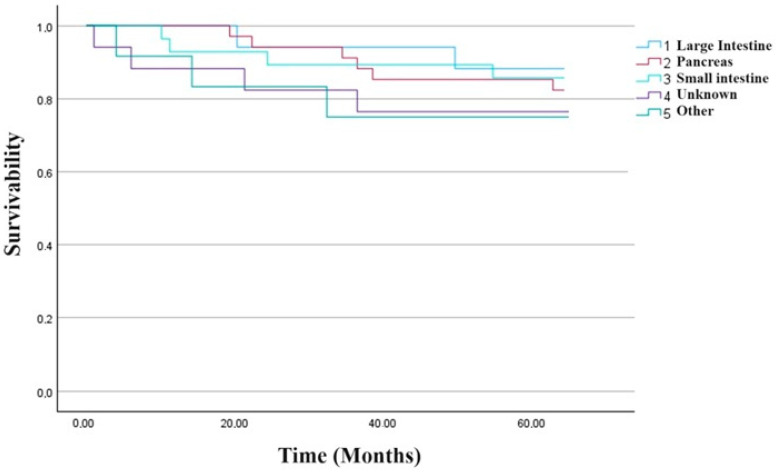
Kaplan–Meyer plot of the study group depending on primary tumor location.

The median observation time in the stabilization subgroup (n = 67) was 29.8 (IQR 22.6). PFS in the progression subgroup (n = 28) was 29.3 (IQR 23.9), and OS in the subgroup with confirmed death (n = 13) was 34.0 (IQR= 16.0). In 19 cases, detailed data were unavailable. Details of the treatment are presented in Table 5 and Figure 9.

Moreover, we compared the results of patients who received [^177^Lu]Lu-DOTA-TATE and [^177^Lu]Lu-DOTA-TATE/[^90^Y]Y-DOTA-TATE; there were no significant differences in OS, PFS, or OTs (Table 6).

## 4. Discussion

Our study presents a typical Polish NEN patient case study who qualifies for RLT: a 60-year-old woman underwent a surgical operation because of a non-functioning tumor of the pancreas three years before. She underwent histopathological confirmation of NEN G2 and monthly injection of lanreotide.

However, this is only a simplified model of patients in Poland, and practitioners must remember that every patient with NEN requires adjusted treatment. The symptoms reported by patients or incidental imaging tests suggest that the presence (or spread) of NEN requires full diagnostics in this direction and even changes in therapeutic decisions.

The gender distribution among the patients participating in this study was reflective of the general Polish and European gender distribution (51.6% female vs. 48.4% male) [36]. The data from other populational analyses confirm that the distribution is similar in almost every part of the globe [37]. The mean age of patients at the time of NEN diagnosis also corresponded to worldwide trends. Data obtained in modern studies confirm that gastroenteropancreatic NEN incidence is rising steadily in every part of the world. However, the distribution of tumor primary location significantly differs by world region. The US database—The Surveillance, Epidemiology and End Results (SEER)—showed that, in the group of 29,664 patients with gastrointestinal NEN diagnosed before 2011, the most common primary location of the disease was the rectum (17.7%), followed by the small intestine (17.3%), the pancreas (7%), the stomach (6%), and the appendix (3.1%) [38]. On the other hand, a retrospective analysis of 5619 NEN-diagnosed patients in Canadian databases (1994–2009) showed utterly different distributions of primary tumor location, presented as follows: small intestine (18.2%), colon (12.9%), rectum (12.3%), and pancreas (9.3%) [39]. The median age at diagnosis for Canadian patients was very similar to that which was observed in the population of the present study (Central European)—60.5 years old (y.o.). These differences observed in the North American population are also visible in European databases, which showed that, even in the moderately diverse European population, there are some differences in primary tumor location distribution.

The region that has a population that is the most similar to the Polish population is Germany. In a German database (1976–2000), there were 2821 identified cases of neuroendocrine neoplasms, with the most prevalent location being the small intestine. The gender distribution in their population (male 45.8% vs. female 54.2%) also corresponded with local population numbers from our study [40]. Northern European countries, like Norway or the UK, showed a different distribution in the NEN primary location. The top five NET sites in Norway were small intestine (26%), lung (21%), colon (8%), rectum (7%), and pancreas (7%). Meanwhile, in the UK, the order was small intestine, appendix, and pancreas. In southern European countries (Greece and Portugal), the population was diagnosed with tumors with the advantage of gastric, pancreatic, and small intestine location [37,41]. Furthermore, the Asian population presented different types of distribution in NEN location. In Taiwan (1996–2008), 2187 NET cases were diagnosed. The gender distribution was uneven (male 62% vs. female 38%), with a mean age of 57.9 years. The most common primary locations were rectum (25.4%), lungs (20%), and stomach (7.4%) [42]. The updated 2021 Taiwan databases showed a gradual increase in NEN prevalence (0.3 per 100,000 in 1996; 1.51 per 100,000 in 2008; 3.162 per 100,000 in 2015), with the primary distribution of NENs location on a very similar level; there was prevalence in the rectum (29.65%) and lungs (17.22%), and a significant increase was noted in the pancreatic location (10.71%) [43]. The other sizeable Asian database was made in Japan and was based on data gathered between 2009 and 2015 [44]. A total number of 33,215 patients (17,485 with NECs and 15,730 NENs) were diagnosed. The number of NECs surpasses the number of NENs. However, some of the “NECs” could be well-differentiated G3 NENs due to the newest terminology and classifications, which were different upon publishing those study results. The most common site of NEN was the rectum (50.9%), followed by the pancreas (13.9%) and the duodenum (9.0%). The age of NEN patients was similar to other databases, at 62.0 years old. Epidemiological data show that the mean age of NEN diagnosis is in the sixth decade of life. However, even 20–30 y.o. patients can be diagnosed with NEN. It has to be remembered that the most common NEN arises as a novo tumor. However, it can be related to some genetically based disorders like neurofibromatosis (NF), MEN-1 syndrome (MEN-1), von Hippel–Lindau (VHL) syndrome, or tuberous sclerosis complex (TSC) [45,46,47,48,49].

The latest neuroendocrine tumor classification and nomenclature changes separated the NEN G3 and NEC groups. They allowed the use of RLT in patients with relatively high grades based on the Ki-67 index but with the simultaneous presence of somatostatin receptors confirmed in functional imaging (^99m^Tc-scintigraphy, ^111^In- scintigraphy or ^68^Ga-PET/CT). This opened another route for treatment among those patients who previously only qualified for chemotherapy [50]. As chemotherapy is less tolerated and more invasive, many modern studies, recommendations, and guidelines advocate for RLT use before this method of treatment [25,51,52]. Despite the local availability of RLT or chemotherapy, we must also remember that the primary method of NEN treatment is surgery (with or without metastasectomy). If operable, the primary tumor and its metastases should be considered for surgery. There are limited contraindications for surgery, such as vascular or neural infiltration of the neoplasm or patient clinical conditions in which safe anesthesia or surgery is impossible, e.g., coagulation disorders or poor cardiac status [53].

The epidemiological data show that most neuroendocrine neoplasms do not have hormonal activity [4,6,54]. The hormonally “active” neoplasms can produce excessive amounts of serotonin causing carcinoid syndrome (flushes, diarrhea), produce glucagon (glucagonomas causing diabetes, necrolytic erythema), gastrin (gastrinomas causing Zollinger–Ellison syndrome, diarrhea), insulin (insulinomas causing hypoglycemia), or vasoactive intestine peptide (VIP-omas, causing diarrhea) [55]. In our analysis, we separated this subgroup to avoid statistical errors in assessing patients with tumors secreting specific types of hormones. It should also be remembered that almost 50% of insulinomas present with no somatostatin analogs receptors. Thus, SSA or RLT will not be effective treatment routes [56,57]. In cases of non-surgical insulinomas, when the presence of somatostatin receptors is not confirmed, the first line of treatment is diazoxide [58]. This might be one reason for our observational study’s lack of insulinoma patients. Some cases of gastrinoma can also be treated with proton pump inhibitors only; however, in disease progression, SSA followed by RLT is necessary. Hence, there is a representative number of gastrinoma patients in the observation [59].

The overwhelming predominance of patients intaking lanreotide vs. octreotide arises from the fact of different registration of the drugs. Lanreotide is registered in midgut gastroenteropancreatic NEN G1 and G2 with Ki-67 <10% and tumors of unknown origin, with non-surgical tumors and metastases. Long-lasting octreotide is registered in midgut NEN and functional NEN of the stomach, intestines, and pancreas. These distinctive differences and the fact that the application of lanreotide in autogel is more accessible and can be performed by the patient themselves or by an educated family member; these are the advantages of this treatment [60,61,62,63]. Ryan et al.’s study compared octreotide and lanreotide, finding no differences in their biochemical outcomes. However, the time of drug administration and easiness of preparation and injection support the use of lanreotide [64]. The mentioned drugs are also registered to treat acromegaly thyrotropinoma (TSH-oma), a specific type of tumor derived from the neuroectoderm. They proved their usefulness and effectiveness in these indications.

The NETTER-1 study compared the effectiveness of SSA with that of RLT. The study showed that RLT with [^177^Lu]Lu-DOTA-TATE compared to 60 mg octreotide a month (a study dose was double the standard) had no significant improvement in the median overall survival (OS). Despite the results showing no statistical significance in OS, there was an 11.7-month difference in median OS with the ^177^Lu-DOTA-TATE group. Almost one additional year in an individual patient’s context must be considered clinically relevant [65]. There is a lack of high-quality double-blind studies comparing monotherapy with [^177^Lu]Lu-DOTA-TATE with tandem [^177^Lu]Lu-DOTA-TATE/[^90^Y]Y-DOTA-TATE, but physical features of ^90^Y cause more concerns in using this type of treatment. A possible higher number of adverse events, like decreasing GFR, liver injury, or bone marrow dysfunction, was not statistically proven, with even some data advocating for higher effectiveness of the method in treating large tumors or metastases [13,15,66,67,68].

In the median two-and-half-year observation after the RLT, we noticed the therapy caused disease stabilization or partial regression in 55.56% of patients’ progression in 26.85%, while only 17.59% died. The median PFS was 29.3 (IQR 23.9), while the median OS was 34.0 months (IQR 16.0). The results obtained during observation did not depend on the radioisotope used for RLT. The study included nearly six years of observation of patients with progressive NEN. In previous studies, however, authors showed divergent results that conformed to specific trends. Brabander et al. analyzed 610 patients treated with a cumulative dose of at least 100 mCi (3.7 GBq) ^177^Lu-DOTA-TATE. In a subgroup of 443 patients treated with a cumulative dose of at least 600 mCi (22.2 GBq) ^177^Lu-DOTA-TATE, they noticed disease stabilization or response in 82% of patients. Progression-free survival (PFS) and overall survival (OS) in the study group were 29 months and 63 months, respectively [69]. Paganelli et al. studied 43 patients who received 3.7 GBq or 5.5 GBq of ^177^Lu-DOTA-TATE. The median observation time was 118 months. Median PFS in patients receiving 18.5 GBq was 59.8 months and did not differ from a subgroup receiving 27.5 GBq. Median OS was 71.0 months in the group treated with 18.5 GBq and 97.6 months in the group who received 27.5 GB. Longer PFS and OS were noticed in subgroups of patients with the disease being limited to the local lymph nodes [70]. In another study, Jiang et al., including a group of 27 patients, noted partial response or disease stabilization in 85.2% of individuals (directly after RLT). The median long-term observation time was 46 months. The median PFS was 36 months, and the median OS was not described. The factor associated with lower PFS was the high initial Ki-67 index (over 10%) [71].

On the other hand, the study mentioned earlier, and one of the most well-known studies concerning neuroendocrine neoplasms—NETTER 1—presented its results, attesting to the effectiveness of treatment with the somatostatin analogs and RLT in 2021. The initial results of 116 patients with well-differentiated metastatic midgut neuroendocrine who received [^177^Lu]Lu-DOTA-TATE at a dose of 7.4 GBq every eight weeks (four intravenous infusions) in addition to the best supportive care—including octreotide long-acting repeatable (LAR) treatment—were compared to a group of 113 patients treated with octreotide alone (at a dose of 60 mg every four weeks) (control group (*p* = 0.004). During primary analysis, PFS at month 20 was 65.2% in the [^177^Lu]Lu-DOTA-TATE group and 10.8% in the octreotide group [72]. It is worth noting that the median PFS at the time of this analysis was 10.5 months (range 0–29 months). The final results of the study (231 patients with a median follow-up of 76 months) showed median overall survival (OS) of 48 months in the [^177^Lu]Lu-DOTA-TATE group and 36.3 months in the octreotide group [65]. Some differences between our results and the findings from other studies of homogeneous NEN groups are noticeable. It may result from different types of NEN qualified for RLT and the initial stage of the disease.

Moreover, our study group was heterogeneous in terms of the therapy length (number of cycles), the initial tumor site (GEP NEN and non-GEP NEN), and disease grading (G1–G3). This is because all the patients who progressed on previous treatment (SSA, surgery) or did not meet the eligibility criteria for other forms of treatment were included. The common factor of treatment qualification was NEN with expression of somatostatin receptors on tumor cells confirmed in ^99m^Tc-scintigraphy or [^68^Ga]Ga-PET/CT.

Hypertension is the most common disease of the cardiovascular system. The populational prevalence of the disease correlates with age and is very similar in modern countries. Epidemiological data show that almost 60% of the population over the sixth decade of life have hypertension [73,74,75]. Our study results match trends, as in the study group; over half of the patients had a diagnosis of hypertension. Data from single-center observation of NEN patients suggest that there might be some influence of RLT on the worsening of blood pressure control [76]. However, there is a need for clear studies that focus on that problem; hence, it requires further observation. Diabetes is a drastically increasing problem in developed and developing countries [77,78,79]. Primarily due to lack of exercise and improper diet, obesity leads to hyperinsulinemia, insulin resistance, and type 2 diabetes. In our study group, 44% of patients had prediabetes or diabetes diagnosed before RLT. The possible diabetogenic action of SSA was confirmed in some studies, but the influence of potential RLT on glycemia remains unclear [80,81]. Hyperlipidemia also globally contributes to an increased number of cardiovascular complications; due to elevated concentrations of some cholesterol fractions, the probability of atherosclerosis and its outcomes (coronary disease, stroke) increases [82,83]. The diagnosis of hyperlipidemia was confirmed in a little over 1/3 of patients (n = 61) in our study. However, the disturbing fact was that 50 patients had all three analyzed comorbidities.

The treatment of RLT in Polish conditions is highly limited by national insurance payments. Because the law monopolizes high-value procedures, the National Insurance Fund (National Health Fund—NHF) controls the number of patients and available therapies. Due to inadequate funding in the analyzed period, not all regional NEN Centers could conduct therapy using ^177^Lu and ^90^Y. In the analyzed years, due to the Ethical Committee Agreement, the Department of Endocrinology and Radioisotope Therapy of the Military Institute of Medicine could provide undisturbed treatment for patients from all over the country. Previously, in 2015, in accordance with the decision made by the European Neuroendocrine Tumor Society (ENETS), the first European Excellence Center was set up in Poland (in the Department of Endocrinology and Neuroendocrine Tumor, Medical University of Silesia, Katowice). After that, other centers joined, and a nationwide network of specialists concerning NEN treatment started bi-weekly online meetings, where patient cases were discussed. These periodic multicenter meetings allow for the coordination of the national treatment of patients with NENs. At the same time, regional centers act as specialist hubs, where patients can be referred to the Excellence Center, which provides the best lines of treatment. This is also the reason for the local disproportion of patients referred for RLT presented in the map in Figure 8 as the leading centers are located in Poland’s Silesian and Mazovian regions. Thanks to the gained experience and undisrupted cooperation, the highest therapy standards that are tailored to modern guidelines were created. The results were published in 2017 and updated in 2022 as guidelines in NEN treatment and diagnosis [4]. Thanks to the exchange of experiences mentioned above, the benefit for patients, as well as for medical professionals and healthcare system, is undeniable. This type of cooperation should be considered in other countries, as it could benefit local populations and medical professionals.

The worldwide increasing incidence and prevalence of neuroendocrine neoplasms poses new challenges for physicians and healthcare systems. NENs are an extremely heterogeneous group, so there is a high need for individual approaches and personalized treatment plans. Developing new and more accurate diagnostic and therapeutic methods will probably increase the number of patients requiring medical attention and long-term care. There is a need to focus more on the problem of NENs in the future, in both medical and patient groups. Moreover, there needs to be more accurate epidemiological data in the literature explaining what kind of patients should be monitored or tested more frequently to improve their overall survival rate. Radioligand therapy remains an unexplored treatment method, and there are no established data surrounding its efficiency in NEN patients. We still need more data proving what kind of patients could benefit from faster RLT qualification, so further observations and analyses are needed.

## 5. Conclusions

Among the participants included in the present study, neuroendocrine neoplasms were diagnosed in the sixth decade of life, and the average time from tumor diagnosis to radioligand therapy was approximately three years. RLT leads to disease stabilization in over half of the patients with progressive disease in long-term observation, thus offering a valid treatment option. There were no differences in overall or progression-free survival depending on the radioisotope used for RLT. Organized coordination of NEN treatment in high-reference Excellence Centers ensures continuity in patient care. Future studies should also focus on identifying those patients who might benefit from RLT at an earlier stage.

## 6. Study Limitations

The analyzed data were collected during the SARS-CoV-2 pandemic, limiting the country’s treatment and diagnostics availability.

## 7. Study Strengths

The study was a prospective one. Data were based on the most significant population of NEN patients treated with RLT in Poland. The authors’ center was the only one that kept this treatment available in the study period.

## Figures and Tables

**Table 1:** Detailed age analysis during diagnosis and first RLT.

	Age	Female (n = 85)	Male (n = 82)	Total (n = 167)
Diagnosis	M	56.95	57.51	57.23
	SD	13.51	11.81	12.69
Treatment	M	59.80	60.01	59.90
	SD	12.85	11.83	12.36

M—mean; SD—standard deviation; RLT—radioligand therapy.

**Table 2:** Medians (Med.) and interquartile ranges (IQR) of chromogranin A (CgA) in functioning and non-functioning tumors.

	CgA before RLT		CgA after RLT		*p*
	Median	IQR	Median	IQR	
Carcinoid (n = 33)	466.0	5050.6	383.5	7840.8	0.509
* Functioning (n = 43)	298.1	1941.7	227.6	5425.8	0.659
Non-functioning (n = 79)	96.9	240.1	82.8	187.6	0.018
Total (n = 122)	133.1	359.4	102.9	395.0	0.034

* Functioning—includes carcinoid cases and other functioning tumors like insulinoma, gastrinoma, etc.; [CgA normal range: 19–100 ng/mL].

**Table 3:** A detailed description of the non-surgical group (n—number of patients; y.o.—years old).

No Surgery	Feature	Results
Age	M	61.8 y.o.
	SD	10.2 y.o.
Gender	Female	n = 25
	Male	n = 26
Location	pancreas	n = 20
	unknown	n = 20
	other	n = 7
	small intestine	n = 4

**Table 4:** Detailed data of chemotherapy used previous to RLT.

I	capecitabine + temozolomide	CAPTEM	n = 7
	etoposide + cisplatin	E/P	n = 6
	everolimus	EVR	n = 5
	unknown	UNK	n = 4
	sunitinib	SU	n = 2
	capecitabine + oxaliplatin	XELOX	n = 2
	5-fluorouracil	5FU	n = 1
	5-fluorouracil + leucovorin	5FU/LV	n = 1
	gemcitabine	GEM	n = 1
	cisplatin + vinorelbine	PN	n = 1
	folinic acid, fluorouracil and irinotecan	FOLFIRI	n = 1
	Etoposide + carboplatin	E/CP	n = 1
II	docetaxel	DTX	n = 1
	5-fluorouracil + leucovorin	5FU/LV	n = 1
III	capecitabine + temozolomide	CAPTEM	n = 1

I—first line of chemotherapy; II—second line of chemotherapy; III—third line of chemotherapy; n—number of patients.

**Table 5:** Medians and interquartile range regarding treatment outcomes in months.

	n	Med. [Months]	IQR
OS	13	34.0	16.0
PFS	28	29.3	23.9
OT_S_	67	29.8	22.6
UNK	19	NA	

OS—overall survival; PFS—progression-free survival; OT_S_—observation time in the subgroup with stabilization; UNK—unknown; NA—not applicable; n = number; Med.—median; IQR—interquartile range.

**Table 6:** Compared results of OS, PFS, and OTs in [^177^Lu]Lu-DOTA-TATE and [^177^Lu]Lu-DOTA-TATE/[^90^Y]Y-DOTA-TATE subgroups.

Radioisotope	[^177^Lu]Lu-DOTA-TATE (n = 99)			[^177^Lu]Lu-DOTA-TATE/[^90^Y]Y-DOTA-TATE (n = 28)			
Parameter	n	Med.	IQR	n	Med.	IQR	*p*
OS	10	30.0	21.5	3	34.0	15.0	0.469
PFS	22	29.3	21.1	34	28.2	27.7	0.935
OTs	54	29.8	21.1	15	29.2	27.7	0.868

OS—overall survival; PFS—progression-free survival; OT_S_—observation time in the subgroup with stabilization; n = number; Med.—median; IQR—interquartile range.

## Data Availability

Data other than that published in the manuscript are partially unavailable due to privacy or ethical restrictions.

## References

[1] Leotlela P.D., Jauch A., Holtgreve-Grez H., Thakker R.V. (2003). Genetics of neuroendocrine and carcinoid tumours. Endocr.-Relat. Cancer.

[2] Raphael M.J., Chan D.L., Law C., Singh S. (2017). Principles of diagnosis and management of neuroendocrine tumours. Can. Med. Assoc. J..

[3] Rindi G., Mete O., Uccella S., Basturk O., La Rosa S., Brosens L.A.A., Ezzat S., de Herder W.W., Klimstra D.S., Papotti M. (2022). Overview of the 2022 WHO Classification of Neuroendocrine Neoplasms. Endocr. Pathol..

[4] Kos-Kudła B., Foltyn W., Malczewska A., Bednarczuk T., Bolanowski M., Borowska M., Chmielik E., Ćwikła J.B., Gisterek I., Handkiewicz-Junak D. (2022). Update of the diagnostic and therapeutic guidelines for gastro-entero-pancreatic neuroendocrine neoplasms (recommended by the Polish Network of Neuroendocrine Tumours). Endokrynol. Pol..

[5] Spigel D.R., Hainsworth J.D., Greco F.A. (2009). Neuroendocrine Carcinoma of Unknown Primary Site. Semin. Oncol..

[6] Yao J.C., Hassan M.M., Phan A.T., Dagohoy C.G., Leary C.C., Mares J.E., Abdalla E.K., Fleming J.B., Vauthey J.-N., Rashid A. (2008). One hundred years after “carcinoid”: Epidemiology of and prognostic factors for neuroendocrine tumors in 35,825 cases in the United States. J. Clin. Oncol..

[7] Juhlin C.C., Zedenius J., Höög A. (2022). Metastatic Neuroendocrine Neoplasms of Unknown Primary: Clues from Pathology Workup. Cancers.

[8] Urso L., Nieri A., Rambaldi I., Castello A., Uccelli L., Cittanti C., Panareo S., Gagliardi I., Ambrosio M.R., Zatelli M.C. (2022). Radioligand therapy (RLT) as neoadjuvant treatment for inoperable pancreatic neuroendocrine tumors: A literature review. Endocrine.

[9] Kolasińska-Ćwikła A., Łowczak A., Maciejkiewicz K.M., Ćwikła J.B. (2018). Peptide Receptor Radionuclide Therapy for Advanced Gastroenteropancreatic Neuroendocrine Tumors—From oncology perspective. Nucl. Med. Rev. Cent. East Eur..

[10] Piwowarska-Bilska H., Kurkowska S., Birkenfeld B. (2022). Optimized method for normal range estimation of standardized uptake values (SUVmax, SUVmean) in liver SPECT/CT images with somatostatin analog [99mTc]-HYNIC-TOC (Tektrotyd). Nucl. Med. Rev. Cent. East Eur..

[11] Paiella S., Landoni L., Tebaldi S., Zuffante M., Salgarello M., Cingarlini S., D’Onofrio M., Parisi A., Deiro G., Manfrin E. (2022). Dual-Tracer (68Ga-DOTATOC and 18F-FDG-)-PET/CT Scan and G1-G2 Nonfunctioning Pancreatic Neuroendocrine Tumors: A Single-Center Retrospective Evaluation of 124 Nonmetastatic Resected Cases. Neuroendocrinology.

[12] Chen S.-H., Chang Y.-C., Hwang T.-L., Chen J.-S., Chou W.-C., Hsieh C.-H., Yeh T.-S., Hsu J.-T., Yeh C.-N., Tseng J.-H. (2018). 68Ga-DOTATOC and 18F-FDG PET/CT for identifying the primary lesions of suspected and metastatic neuroendocrine tumors: A prospective study in Taiwan. J. Formos. Med. Assoc..

[13] Sowa-Staszczak A., Pach R., Kunikowska J., Krolicki L., Stefanska A., Tomaszuk M., Buziak-Bereza M., Mikolajczak R., Matyja M., Gilis-Januszewska A. (2011). Efficacy and safety of 90Y-DOTATATE therapy in neuroendocrine tumours. Endokrynol. Pol..

[14] Bodei L., Kidd M., Paganelli G., Grana C.M., Drozdov I., Cremonesi M., Lepensky C., Kwekkeboom D.J., Baum R.P., Krenning E.P. (2015). Long-term tolerability of PRRT in 807 patients with neuroendocrine tumours: The value and limitations of clinical factors. Eur. J. Nucl. Med. Mol. Imaging.

[15] Kunikowska J., Królicki L., Hubalewska-Dydejczyk A., Mikołajczak R., Sowa-Staszczak A., Pawlak D. (2011). Clinical Results of Radionuclide Therapy of Neuroendocrine Tumours with 90Y-DOTATATE and Tandem 90Y/^177^LuDOTATATE: Which Is a Better Therapy Option?. Eur. J. Nucl. Med. Mol. Imaging.

[16] Ramage J., De Herder W., Fave G.D., Ferolla P., Ferone D., Ito T., Ruszniewski P., Sundin A., Weber W., Zheng-Pei Z. (2016). ENETS Consensus Guidelines Update for Colorectal Neuroendocrine Neoplasms. Neuroendocrinology.

[17] Pavel M., Öberg K., Falconi M., Krenning E.P., Sundin A., Perren A., Berruti A., ESMO Guidelines Committee (2020). Gastroenteropancreatic neuroendocrine neoplasms: ESMO Clinical Practice Guidelines for diagnosis, treatment and follow-up. Ann. Oncol..

[18] Öberg K., Castellano D. (2011). Current knowledge on diagnosis and staging of neuroendocrine tumors. Cancer Metastasis Rev..

[19] Luchini C., Pantanowitz L., Adsay V., Asa S.L., Antonini P., Girolami I., Veronese N., Nottegar A., Cingarlini S., Landoni L. (2022). Ki-67 assessment of pancreatic neuroendocrine neoplasms: Systematic review and meta-analysis of manual vs. digital pathology scoring. Mod. Pathol..

[20] Farrell J.M., Pang J.C., Kim G.E., Tabatabai Z.L. (2014). Pancreatic neuroendocrine tumors: Accurate grading with Ki-67 index on fine-needle aspiration specimens using the WHO 2010/ENETS criteria. Cancer Cytopathol..

[21] Tacelli M., Bina N., Crinò S.F., Facciorusso A., Celsa C., Vanni A.S., Fantin A., Antonini F., Falconi M., Monica F. (2022). Reliability of grading preoperative pancreatic neuroendocrine tumors on EUS specimens: A systematic review with meta-analysis of aggregate and individual data. Gastrointest. Endosc..

[22] Heidsma C.M., Tsilimigras D.I., Rocha F., Abbott D.E., Fields R., Smith P.M., Poultsides G.A., Cho C., van Eijck C., van Dijkum E.N. (2020). Clinical relevance of performing endoscopic ultrasound-guided fine-needle biopsy for pancreatic neuroendocrine tumors less than 2 cm. J. Surg. Oncol..

[23] Kawasaki Y., Hijioka S., Nagashio Y., Maruki Y., Ohba A., Takeshita K., Takasaki T., Agarie D., Hagiwara Y., Hara H. (2023). Efficacy of endoscopic ultrasound-guided tissue acquisition for solid pancreatic lesions 20 mm or less in diameter suspected as neuroendocrine tumors or requiring differentiation. J. Gastroenterol..

[24] Mastrosimini M.G., Manfrin E., Remo A., De Bellis M., Parisi A., Pedron S., Luchini C., Brunelli M., Ammendola S., Bernardoni L. (2023). Endoscopic ultrasound fine-needle biopsy to assess DAXX/ATRX expression and alternative lengthening of telomeres status in non-functional pancreatic neuroendocrine tumors. Pancreatology.

[25] Baudin E., Gigliotti A., Ducreux M., Ropers J., Comoy E., Sabourin J., Bidart J., Cailleux A., Bonacci R., Ruffié P. (1998). Neuron-specific enolase and chromogranin A as markers of neuroendocrine tumours. Br. J. Cancer.

[26] Fuksiewicz M., Kowalska M., Kolasińska-Ćwikła A., Ćwikła J.B., Sawicki Ł., Roszkowska-Purska K., Drygiel J., Kotowicz B. (2018). Prognostic value of chromogranin A in patients with GET/NEN in the pancreas and the small intestine. Endocr. Connect..

[27] Dąbkowski K., Starzyńska T. (2023). Management of small, asymptomatic, non-functioning pancreatic neuroendocrine tumours: Follow-up, ablation, or surgery?. Endokrynol. Pol..

[28] Crinò S.F., Napoleon B., Facciorusso A., Lakhtakia S., Borbath I., Caillol F., Pham K.D.-C., Rizzatti G., Forti E., Palazzo L. (2023). Endoscopic Ultrasound-guided Radiofrequency Ablation Versus Surgical Resection for Treatment of Pancreatic Insulinoma. Clin. Gastroenterol. Hepatol..

[29] Elkelany O.O., Karaisz F.G., Davies B., Krishna S.G. (2023). An Overview of Pancreatic Neuroendocrine Tumors and an Update on Endoscopic Techniques for Their Management. Curr. Oncol..

[30] Armellini E., Facciorusso A., Crinò S.F. (2023). Efficacy and Safety of Endoscopic Ultrasound-Guided Radiofrequency Ablation for Pancreatic Neuroendocrine Tumors: A Systematic Review and Metanalysis. Medicina.

[31] Ebbers S.C., Braat A.J.A.T., Moelker A., Stokkel M.P.M., Lam M.G.E.H., Barentsz M.W. (2020). Intra-arterial versus standard intravenous administration of lutetium-177-DOTA-octreotate in patients with NET liver metastases: Study protocol for a multicenter, randomized controlled trial (LUTIA trial). Trials.

[32] Chan D.L., Singh S. (2018). Current Chemotherapy Use in Neuroendocrine Tumors. Endocrinol. Metab. Clin. N. Am..

[33] Bardasi C., Spallanzani A., Benatti S., Spada F., Laffi A., Antonuzzo L., Lavacchi D., Marconcini R., Ferrari M., Rimini M. (2021). Irinotecan-based chemotherapy in extrapulmonary neuroendocrine carcinomas: Survival and safety data from a multicentric Italian experience. Endocrine.

[34] Andreetti C., Ibrahim M., Gagliardi A., Poggi C., Maurizi G., Armillotta D., Peritone V., Teodonio L., Rendina E.A., Venuta F. (2022). Adjuvant chemotherapy, extent of resection, and immunoistochemical neuroendocrine markers as prognostic factors of early-stage large-cell neuroendocrine carcinoma. Thorac. Cancer.

[35] Oziel-Taieb S., Zemmour C., Raoul J.-L., Mineur L., Poizat F., Charrier N., Piana G., Cavaglione G., Niccoli P. (2021). Efficacy of FOLFOX Chemotherapy in Metastatic Enteropancreatic Neuroendocrine Tumors. Anticancer. Res..

[36] Cierniak-Piotrowska M., Dąbrowska A., Stelmach K., Statistics Poland, Demographic Surveys Department Population. Size and Structure and Vital Statistics in Poland by Territorial Division in 2022. As of 30 June. https://stat.gov.pl/en/topics/population/population/population-size-and-structure-and-vital-statistics-in-poland-by-territorial-division-in-2022-as-of-30-june-2022,3,32.html.

[37] Das S., Dasari A. (2021). Epidemiology, Incidence, and Prevalence of Neuroendocrine Neoplasms: Are There Global Differences?. Curr. Oncol. Rep..

[38] Lawrence B., Gustafsson B.I., Chan A., Svejda B., Kidd M., Modlin I.M. (2011). The Epidemiology of Gastroenteropancreatic Neuroendocrine Tumors. Endocrinol. Metab. Clin. N. Am..

[39] Hallet J., Law C., Cukier M., Saskin R., Liu N., Singh S. (2015). Exploring the rising incidence of neuroendocrine tumors: A population-based analysis of epidemiology, metastatic presentation, and outcomes. Cancer.

[40] Scherübl H., Streller B., Stabenow R., Herbst H., Höpfner M., Schwertner C., Zappe S.M. (2013). Clinically detected gastroenteropancreatic neuroendocrine tumors are on the rise: Epidemiological changes in Germany. World J. Gastroenterol..

[41] Hauso O., Gustafsson B.I., Kidd M., Waldum H.L., Drozdov I., Chan A.K.C., Modlin I.M. (2008). Neuroendocrine tumor epidemiology. Cancer.

[42] Tsai H.-J., Wu C.-C., Tsai C.-R., Lin S.-F., Chen L.-T., Chang J.S. (2013). The Epidemiology of Neuroendocrine Tumors in Taiwan: A Nation-Wide Cancer Registry-Based Study. PLoS ONE.

[43] Chang J.S., Chen L.-T., Shan Y.-S., Chu P.-Y., Tsai C.-R., Tsai H.-J. (2021). An updated analysis of the epidemiologic trends of neuroendocrine tumors in Taiwan. Sci. Rep..

[44] Koizumi T., Otsuki K., Tanaka Y., Kanda S. (2022). Epidemiology of neuroendocrine neoplasmas in Japan: Based on analysis of hospital-based cancer registry data, 2009–2015. BMC Endocr. Disord..

[45] Gauci J., Azzopardi N., Babic D., Cortis K., Axisa B. (2022). Neurofibromatosis Type 1. Pancreas.

[46] Effraimidis G., Knigge U., Rossing M., Oturai P., Rasmussen K., Feldt-Rasmussen U. (2022). Multiple endocrine neoplasia type 1 (MEN-1) and neuroendocrine neoplasms (NENs). Semin. Cancer Biol..

[47] Binderup M.L.M., Smerdel M., Borgwadt L., Nielsen S.S.B., Madsen M.G., Møller H.U., Kiilgaard J.F., Friis-Hansen L., Harbud V., Cortnum S. (2022). von Hippel-Lindau disease: Updated guideline for diagnosis and surveillance. Eur. J. Med. Genet..

[48] Zwolak A., Świrska J., Tywanek E., Dudzińska M., Tarach J.S., Matyjaszek-Matuszek B. (2020). Pancreatic neuroendocrine tumours in patients with von Hippel-Lindau disease. Endokrynol. Pol..

[49] Evans L.M., Geenen K.R., O’Shea A., Hedgire S.S., Ferrone C.R., Thiele E.A. (2022). Tuberous sclerosis complex-associated nonfunctional pancreatic neuroendocrine tumors: Management and surgical outcomes. Am. J. Med. Genet. Part A.

[50] Sorbye H., Kong G., Grozinsky-Glasberg S. (2020). PRRT in high-grade gastroenteropancreatic neuroendocrine neoplasms (WHO G3). Endocr.-Relat. Cancer.

[51] de Mestier L., Walter T., Evrard C., de Boissieu P., Hentic O., Cros J., Ruszniewski P. (2020). Temozolomide alone or combined with capecitabine for the treatment of advanced pancreatic neuroendo-crinetumor. Neuroendocrinology.

[52] Borga C., Businello G., Murgioni S., Bergamo F., Martini C., De Carlo E., Trevellin E., Vettor R., Fassan M. (2021). Treatment personalization in gastrointestinal neuroendocrine tumors. Curr. Treat. Options Oncol..

[53] Kohno S. (2022). Diagnosis and Surgical Treatment of Gastroenteropancreatic Neuroendocrine Neoplasms: A Literature Review. Cancer Diagn. Progn..

[54] Juhlin C.C., Skoglund S., Juntti-Berggren L., Karlberg M., Calissendorff J. (2019). Non-functioning neuroendocrine pancreatic tumors transforming to malignant insulinomas - four cases and review of the literature. Neuro Endocrinol. Lett..

[55] Fang J.M., Li J., Shi J. (2022). An update on the diagnosis of gastroenteropancreatic neuroendocrine neoplasms. World J. Gastroenterol..

[56] de Herder W.W., Zandee W.T., Hofland J., Feingold K.R., Anawalt B., Blackman M.R., Boyce A., Chrousos G., Corpas E., de Herder W.W., Dhatariya K., Dungan K., Hofland J. (2000). Insulinoma. Endotext.

[57] Portela-Gomes G.M., Stridsberg M., Grimelius L., Rorstad O., Janson E.T. (2007). Differential Expression of the Five Somatostatin Receptor Subtypes in Human Benign and Malignant Insulinomas—Predominance of Receptor Subtype 4. Endocr. Pathol..

[58] Warren A.M., Topliss D.J., Hamblin P.S. (2020). Successful medical management of insulinoma with diazoxide for 27 years. Endocrinol. Diabetes Metab. Case Rep..

[59] Rossi R.E., Elvevi A., Citterio D., Coppa J., Invernizzi P., Mazzaferro V., Massironi S. (2021). Gastrinoma and Zollinger Ellison syndrome: A roadmap for the management between new and old therapies. World J. Gastroenterol..

[60] Broder M.S., Beenhouwer D., Strosberg J.R., Neary M.P., Cherepanov D. (2015). Gastrointestinal neuroendocrine tumors treated with high dose octreotide-LAR: A systematic literature review. World J. Gastroenterol..

[61] Pavel M., Borson-Chazot F., Cailleux A., Hörsch D., Lahner H., Pivonello R., Tauchmanova L., Darstein C., Olsson H., Tiberg F. (2019). Octreotide SC depot in patients with acromegaly and functioning neuroendocrine tumors: A phase 2, multicenter study. Cancer Chemother. Pharmacol..

[62] Lepage C., Phelip J.-M., Lievre A., Le-Malicot K., Dahan L., Tougeron D., Toumpanakis C., Di-Fiore F., Lombard-Bohas C., Borbath I. (2022). Lanreotide as maintenance therapy after first-line treatment in patients with non-resectable duodeno-pancreatic neuroendocrine tumours: An international double-blind, placebo-controlled randomised phase II trial—Prodige 31 REMINET: An FFCD study. Eur. J. Cancer.

[63] Paulson S., Ray D., Aranha S., Scales A., Wang Y., Liu E. (2022). Lanreotide Depot to Treat Gastroenteropancreatic Neuroendocrine Tumors in a US Community Oncology Setting: A Prospective, Observational Study. Oncol. Ther..

[64] Ryan P., McBride A., Ray D., Pulgar S., Ramirez R., Elquza E., Favaro J., Dranitsaris G. (2019). Lanreotide vs octreotide LAR for patients with advanced gastroenteropancreatic neuroendocrine tumors: An observational time and motion analysis. J. Oncol. Pharm. Prat..

[65] Strosberg J.R., Caplin M.E., Kunz P.L., Ruszniewski P.B., Bodei L., Hendifar A., Mittra E., Wolin E.M., Yao J.C., Pavel M.E. (2021). 177Lu-Dotatate plus long-acting octreotide versus high-dose long-acting octreotide in patients with midgut neuroendocrine tumours (NETTER-1): Final overall survival and long-term safety results from an open-label, randomised, controlled, phase 3 trial. Lancet Oncol..

[66] Saracyn M., Durma A.D., Bober B., Kołodziej M., Lubas A., Kapusta W., Niemczyk S., Kamiński G. (2023). Long-Term Complications of Radioligand Therapy with Lutetium-177 and Yttrium-90 in Patients with Neuroendocrine Neoplasms. Nutrients.

[67] Bober B., Saracyn M., Zaręba K., Lubas A., Mazurkiewicz P., Wilińska E., Kamiński G. (2022). Early Complications of Radioisotope Therapy with Lutetium-177 and Yttrium-90 in Patients with Neuroendocrine Neoplasms—A Preliminary Study. J. Clin. Med..

[68] Hörsch D., Ezziddin S., Haug A., Gratz K.F., Dunkelmann S., Miederer M., Schreckenberger M., Krause B.J., Bengel F.M., Bartenstein P. (2016). Effectiveness and side-effects of peptide receptor radionuclide therapy for neuroendocrine neoplasms in Germany: A multi-institutional registry study with prospective follow-up. Eur. J. Cancer.

[69] Brabander T., van der Zwan W.A., Teunissen J.J., Kam B.L., Feelders R.A., de Herder W.W., van Eijck C.H., Franssen G.J., Krenning E.P., Kwekkeboom D.J. (2017). Long-Term Efficacy, Survival, and Safety of [^177^Lu-DOTA0,Tyr3]octreotate in Patients with Gastroenteropancreatic and Bronchial Neuroendocrine Tumors. Clin. Cancer Res..

[70] Paganelli G., Sansovini M., Nicolini S., Grassi I., Ibrahim T., Amadori E., Di Iorio V., Monti M., Scarpi E., Bongiovanni A. (2021). ^177^Lu-PRRT in advanced gastrointestinal neuroendocrine tumors: 10-year follow-up of the IRST phase II prospective study. Eur. J. Nucl. Med. Mol. Imaging.

[71] Jiang Y., Liu Q., Wang G., Sui H., Wang R., Wang J., Zhang J., Zhu Z., Chen X. (2022). Safety and efficacy of peptide receptor radionuclide therapy with ^177^Lu-DOTA-EB-TATE in patients with metastatic neuroendocrine tumors. Theranostics.

[72] Strosberg J., El-Haddad G., Wolin E., Hendifar A., Yao J., Chasen B., Mittra E., Kunz P.L., Kulke M.H., Jacene H. (2017). Phase 3 Trial of ^177^Lu-Dotatate for Midgut Neuroendocrine Tumors. N. Engl. J. Med..

[73] Banegas J., Gijón-Conde T. (2017). Epidemiología de la hipertensión arterial. Hipertens. Riesgo Vasc..

[74] Al Ghorani H., Götzinger F., Böhm M., Mahfoud F. (2022). Arterial hypertension—Clinical trials update 2021. Nutr. Metab. Cardiovasc. Dis..

[75] Hisamatsu T., Segawa H., Kadota A., Ohkubo T., Arima H., Miura K. (2020). Epidemiology of hypertension in Japan: Beyond the new 2019 Japanese guidelines. Hypertens. Res..

[76] Saracyn M., Durma A.D., Bober B., Lubas A., Kołodziej M., Kapusta W., Dmochowska B., Kamiński G. (2023). Renal Disturbances during and after Radioligand Therapy of Neuroendocrine Tumors—Extended Analysis of Potential Acute and Chronic Complications. Int. J. Mol. Sci..

[77] Koye D.N., Magliano D.J., Nelson R.G., Pavkov M.E. (2018). The Global Epidemiology of Diabetes and Kidney Disease. Adv. Chronic Kidney Dis..

[78] Misra A., Gopalan H., Jayawardena R., Hills A.P., Soares M., Reza-Albarrán A.A., Ramaiya K.L. (2019). Diabetes in developing countries. J. Diabetes.

[79] Galicia-Garcia U., Benito-Vicente A., Jebari S., Larrea-Sebal A., Siddiqi H., Uribe K.B., Ostolaza H., Martín C. (2020). Pathophysiology of Type 2 Diabetes Mellitus. Int. J. Mol. Sci..

[80] Cappellani D., Urbani C., Sardella C., Scattina I., Marconcini G., Lupi I., Manetti L., Marcocci C., Bogazzi F. (2018). Diabetes mellitus induced by somatostatin analogue therapy is not permanent in acromegalic patients. Endocrinol. Diabetes Metab..

[81] Teunissen J.J.M., Krenning E.P., de Jong F.H., de Rijke Y.B., Feelders R.A., van Aken M.O., de Herder W.W., Kwekkeboom D.J. (2009). Effects of therapy with [^177^Lu-DOTA0,Tyr3]octreotate on endocrine function. Eur. J. Nucl. Med..

[82] Libby P., Buring J.E., Badimon L., Hansson G.K., Deanfield J., Bittencourt M.S., Tokgözoğlu L., Lewis E.F. (2019). Atherosclerosis. Nat. Rev. Dis. Primers.

[83] Guijarro C., Cosín-Sales J. (2021). LDL cholesterol and atherosclerosis: The evidence. Clin. Investig. Arterioscler..

